# Increased sIL-2Rα leads to obstruction of IL-2 biological function and Treg cells differentiation in SLE patients *via* binding to IL-2

**DOI:** 10.3389/fimmu.2022.938556

**Published:** 2022-09-20

**Authors:** Dan Long, Shujiao Yu, Lu Zhang, Yang Guo, Shumin Xu, Yuting Rao, Zikun Huang, Qing Luo, Junming Li

**Affiliations:** Department of Clinical Laboratory, The First Affiliated Hospital of Nanchang University, Nanchang, China

**Keywords:** sIL-2R, IL-2, Treg cells, immune complex, systemic lupus erythematosus

## Abstract

**Background:**

The decrease of IL-2 level is believed to play an important role in the disease occurrence and development of SLE, but the relevant mechanisms have not been fully clarified. Many studies have found that the level of soluble interleukin 2 receptor α (sIL-2Rα) in SLE patients is significantly increased. Considering the fact that sIL-2Rα has the ability to bind IL-2, we want to know whether the increased sIL-2Rα has some impact on the level and function of IL-2 in SLE patients.

**Methods:**

New onset SLE patients, treated SLE patients and healthy volunteers were recruited. The levels of serum IL-2, IL-2 mRNA in CD3^+^ T cells and serum sIL-2Rα were detected and compared in these subjects. Two mixed solid-phase sandwich ELISA system were designed to measure exclusively the heterodimers complex of sIL-2Rα/IL-2. The sera from SLE patients were pretreated with or without immune complex dissociation solution and detected for IL-2 levels. IL-2 standard or serum from HCs were used to co-incubate with recombinant sIL-2Rα or serum samples with high levels of sIL-2Rα and detected for IL-2 levels by ELISA. The inhibitory effect of sIL-2Rα on IL-2 biological activity was investigated by CTLL-2 cell proliferation assay. The frequencies and absolute counts of Treg cells were detected by flow cytometry before and after the addition of recombinant sIL-2Rα.

**Results:**

The levels of serum IL-2 in SLE patients were significantly decreased and negatively correlated with SLEDAI. However, there was no significant difference in IL-2 mRNA levels in CD3^+^ T cells between SLE patients and healthy controls. The levels of serum sIL-2Rα in SLE patients were significantly increased, positively correlated with the SLEDAI and negatively correlated with the levels of serum IL-2. sIL-2Rα was shown to bind to IL-2 to form immune complex, resulting in false reduction in the detection level of serum IL-2 and significant decrease in biological activity of IL-2. The increase of sIL-2Rα was demonstrated to be one of the important mechanisms for the obstruction of Treg cells differentiation in SLE patients.

**Conclusion:**

Increased serum sIL-2Rα can bind to IL-2, leading to obstruction of IL-2 activity and Treg cells differentiation.

## Introduction

Systemic lupus erythematosus (SLE) is one of the most common autoimmune diseases in the world (1). Since the pathogenesis has not been fully clarified, SLE cannot be cured at present (2). Most patients need to maintain the treatment of hormones or immunosuppressants for a long time or even for life. Further clarifying the occurrence and development mechanism of SLE and exploring new therapeutic targets and strategies for SLE have been the direction of researchers in this field. Studies have suggested that genetic, immune and environmental factors jointly affect the occurrence and development of SLE, and abnormal immune regulation is one of the key factors in the development of SLE (3). A large number of studies have shown that one of the main characteristics of SLE patients is the abnormal differentiation and function of T lymphocytes (4). Among them, the decrease in the frequencies and dysfunction of regulatory T (Treg) cells are one of important mechanisms for the compromise of peripheral tolerance and development of SLE (5, 6).

Interleukin-2 (IL-2) is a pleiotropic cytokine required for T cell proliferation and differentiation (7). In particular, it plays a key role in the survival and expansion of Treg cells (8). As a result, IL-2 was recognized as a growth factor of Treg cells (9). Researchers have long observed that the detection level of serum IL-2 in SLE patients is significantly lower than that of healthy people (10). Therefore, the researchers speculated that the decrease of IL-2 level may play a role in the occurrence and development of SLE. Subsequent studies also confirmed this hypothesis (11). Mouse models of SLE support the concept that the decrease of IL-2 level can significantly inhibit the proliferation and differentiation of Treg cells in SLE patients, resulting in the imbalance of immune response and the development of SLE (11). IL-2 complementary therapy based on this finding has gradually attracted attention in the treatment of SLE in recent years and has achieved good therapeutic effects in many clinical trials (12, 13).

However, due to the poor stability of IL-2 and side effects in human application, the scheme of IL-2 supplementation therapy in the treatment of SLE may be not the best scheme (14). Exploring the mechanisms of the decrease of IL-2 level and function in patients with SLE and exploring how to prevent or reverse the decline of IL-2 may be a better method for the treatment of SLE. However, the mechanism of IL-2 decline in SLE patients has not been fully clarified.

Soluble interleukin-2 receptor α (sIL-2Rα) is the soluble form of interleukin-2 receptor (IL-2R) alpha subunit, which is considered to be derived from proteolytic cleavage of membrane IL-2R (15). Literature reports and our previous studies showed that the level of sIL-2Rα in SLE patients was significantly up-regulated (16). Studies in patients with tumors and hematological diseases showed that sIL-2Rα retained the ability to bind IL-2 after its release from cell membrane (17). In this study, we investigated the effects of elevated sIL-2Rα in SLE patients on IL-2 detection and function, as well as the proliferation of Treg cells.

## Materials and methods

### Subjects and study design

From November 2019 to September 2020, patients who met the American College of Rheumatology (ACR) 1997 revised criteria of SLE (18) were recruited from The First Affiliated Hospital of Nanchang University. Disease activity was assessed on the basis of SLE disease activity index (SLEDAI) (19). SLE patients fell into stable-disease cohort (SLEDAI, 0-9) and active-disease cohort (SLEDAI, ≥10) in regard to disease activity. Ninety-nine age- and sex-matched healthy volunteers who were not related to SLE patients and free from inflammation or autoimmune diseases were recruited as HCs. The research protocol was approved by the Ethics Committee of The First Affiliated Hospital of Nanchang University [approval no. (2022)CDYFYYLK(06-005)] and complied with the Helsinki Declaration. All subjects participating in this study provided written informed consent.

### Assay of sIL-2Rα and IL-2

Blood samples were collected into sterile tubes at the time of diagnosis. After centrifugation, the serum was stored at -80°C prior to assay. sIL-2Rα concentration in serum was determined with an Immulite 1000 immunoassay analyzer (Siemens Healthcare Diagnostics, Berlin, Germany), which uses enzyme-amplified chemiluminescent immunoassay technology.

IL-2 in serum was determined using commercially available enzyme-linked immunosorbent assay (ELISA) kits (R&D Systems). This assay recognizes natural and recombinant human IL-2. The researchers who performed these tests were blinded to all the clinical data.

### CD3^+^ T cells collection and RNA extraction

PBMCs were isolated from all subject’s EDTA anticoagulated blood samples using Ficoll-Hypaque density gradients (Sigma-Aldrich; Merck KGaA) within 4 h of collection of the samples. CD3^+^ T cells were purified using human CD3 Mircobeads (Miltenyi Biotec, Bergisch Gladbach, Germany). According to the manufacturer’s protocol, total RNA was extracted from CD3^+^ T cells specimens using TRIzol^®^ (Invitrogen; Thermo Fisher Scientific, Inc.) and stored at -80˚C. The concentration of RNA was quantified and qualified by absorbance spectrometry, the absorbance ratios of A260/A280 and A260/A230 were measured by a NanoDrop ND-1000 spectrophotometer (Agilent Technologies, Inc.).

### Reverse transcription-quantitative PCR analysis

Total RNA from isolated CD3^+^ T cells was reverse transcribed into cDNA using a PrimeScript™ RT reagent kit (Takara Bio, Inc.). The qPCR analysis was carried out on an ABI 7500 Real-time PCR system (Applied Biosystems; Thermo Fisher Scientific, Inc.), using SYBR^®^ Premix Ex Taq™ II (Takara Bio, Inc.). The PCR amplification was performed as follows for 40 cycles: 95˚C for 15 s (denaturation), 60˚C for 1 min (annealing and elongation) and 72˚C for 2 min (final extension). All samples were detected in triplicate. The comparative threshold cycle (Ct) method was used to analyze the data. GAPDH was used as an internal control, and the relative expression of IL-2 was analyzed using the 2-ΔΔCt method. IL-2 primer sequences were: forward 5’-ACCAGGATGCTCACATTTAAGTTTT-3’, reverse 5’-GAGGTTTGAGTTCTTCTTCTAGACACTG -3’.

### Sandwich ELISA for detection of sIL-2Rα/IL-2 complexes

To measure exclusively the heterodimers complex of sIL-2Rα/IL-2, an ELISA specific for the heterodimeric sIL-2Rα/IL-2 was developed as follows: The human anti-IL-2 antibody (R&D Systems) was coated (0.2 μg/ml) on 96-well ELISA plates (BD Biosciences) and incubated overnight at 4°C. After coating, the plates were washed with PBST (PBS containing 0.05% Tween-20) for three times. PBS containing 1% BSA (PBSA, Sigma) was added to each well as blocking buffer. The plates were incubated for 1 h at RT and then washed three times with PBST. Serum from SLE patients or healthy controls was used as samples. 100 μl of standard, control, or sample were added to the rmIL-2-coated wells. Then, 200 μl of 0.4 ug/ml human IL-2R alpha biotinylated antibody (R&D Systems) was added to each well and incubated for 2 h at RT after mixing. After washing three times with PBST, streptavidin-HRP (R&D Systems) was added at a dilution of 1:200. After washing to remove unbound reagents, 200 μl of TMB substrate solution (Thermo Fisher Scientific) was added to the wells and 50 μl stop solution (R&D Systems) was used to stop the color reaction. The absorbance at 450nm was measured using a microplate reader (Bio-Rad, Hercules, CA) to determine the OD value. The results were expressed or plotted as the average of duplicates.

At the same time, this research also carried out reverse verification. The process of enzyme-linked immunosorbent assay is described above. After coating with human IL-2R alpha antibody (R&D Systems), plates were washed, and then standard, control, or sample were added to the rmIL-2Rα-coated wells. Next, 200 μl of 0.4 ug/ml human IL-2 biotinylated antibody (R&D Systems) was added to each well and incubated for 2 h at RT after mixing. The other steps after that are as described above.

### Preparation and application of immune complex dissociation solution

0.3% Triton-X100, 1.5% 3-[(3-cholamidopropyl)-dimethylammonio] propanesulfonic acid (CHAPS) and 1.5% sodium dodecyl sulfate (SDS) are respectively prepared by volume percentage concentration of aqueous solution, according to the volume ratio of 1:1:1, and the mixed liquid (20) was poured into the reagent bottle and saved in 5ml equal parts.

### Detection of biological activity of IL-2

The CTLL-2 cells were cultured at 37˚C in an atmosphere containing 5% CO_2_ in RPMI 1640 complete culture medium supplemented with 10% FBS, 2mM Glutamine and 6 pg/ml Human Recombinant IL-2. The assay of IL-2 activity was conducted using the 3-(4,5-dimethylthiazol-2-yl)-2,5-dimethyltetrazolium bromide (MTT) colorimetric assay, which is based on the mitochondrial dehydrogenase of intact cells that reduces MTT to purple forma nitrogen product (21). Briefly, CTLL-2 cells with good growth conditions were washed with RPMI-1640 medium for 2-3 times and cultured in serum-free and IL-2-free culture medium for 90 min to starve the cells, then supplemented with 10% FBS, 2mM Glutamine and IL-2 (6 pg/ml), followed by treatment with recombinant human sIL-2Rα (R&D Systems, Cat. No. 223-2A-005) at final concentrations of 0, 10, 25, 50, 100, 250, 500, 1×10^3^, 5×10^3^, 1×10^4^, 2.5×10^4^, and 5×10^4^ pg/ml, or ConA (10 μg/ml; Sigma-Aldrich) for 20 hours. CTLL-2 cells cultured without IL-2 were used as the background. During the last 4 hours of the cell culture, MTT solution (Sigma-Aldrich, St. Louis, MO, USA) was added into each well to the final concentration of 0.75 μg/μl. After additional incubation for 4 hours, the medium was removed and 200 μl of dimethyl sulfoxide (DMSO) was added to each well and shaken horizontally for 10 min. The absorbance of the samples was measured at 570 nm using an ELISA plate reader (Bio-Rad, Hercules, CA).

### Flow cytometry detection

The cells were washed twice with PBS and stained for 30 min at room temperature in the dark with fluorescent-labeled anti-human monoclonal antibodies mixture: CD4-fluorescein isothiocyanate (FITC), CD25-PC5, and CD127-PE (Beckman Coulter, Brea, CA, United States). Quantification beads (Beckman Coulter, Brea, CA, United States) were used to assess absolute counts of cell subsets. Background fluorescence and nonspecific staining were assessed using appropriate isotype- and fluorochrome-matched control monoclonal antibodies. The frequencies and absolute counts of CD4^+^CD25^high^CD127^-/low^ Tregs and the percentage of Tregs on gated CD4^+^ lymphocytes were assessed by a Cytomics FC 500 flow cytometer (Beckman Coulter, Brea, CA, United States).

## Results

### IL-2 protein levels, but not mRNA levels are significantly decreased in SLE patients

**Figure 1:**
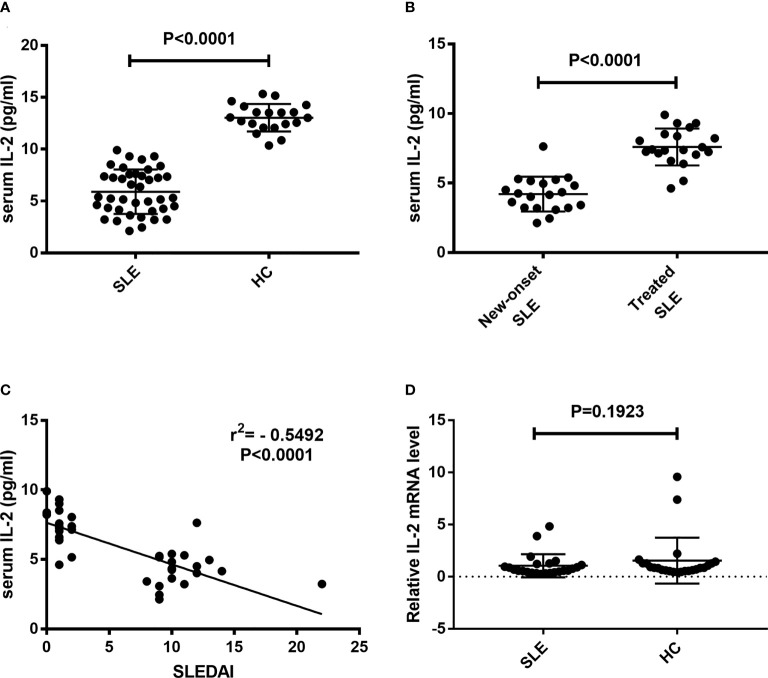
Detection of IL-2 levels in peripheral blood of SLE patients. Serum IL-2 levels and relative IL-2 mRNA levels in CD3^+^ T cells were detected and compared between SLE patients and HCs. **(A)** Serum IL-2 levels in SLE patients was significantly decreased than those in HCs. **(B)** Serum IL-2 levels in new-onset SLE patients was significantly decreased than those in treated SLE patients. **(C)** A negative correlation was observed between serum IL-2 levels and SLEDAI. **(D)** No significant difference was found in the IL-2 mRNA levels of CD3^+^ T cells between SLE patients and HCs. SLE, systemic lupus erythematosus; HCs, healthy controls; SLEDAI, Systemic Lupus Erythematosus Disease Activity Index.

Sera and CD3^+^ T cells were isolated from peripheral blood of SLE patients and healthy controls. Serum IL-2 levels and IL-2 mRNA levels in CD3^+^ T cells were detected. Results showed that the levels of serum IL-2 in SLE patients were significantly decreased than those in HC controls (5.90 ± 0.34 pg/ml *vs.* 13.03 ± 0.30 pg/ml, P<0.0001) (**Figure 1A**). The levels of serum IL-2 in new-onset SLE patients were significantly decreased than those in treated SLE patients (4.21 ± 0.28 pg/ml *vs*. 7.59 ± 0.30 pg/ml, P<0.0001) (**Figure 1B**). In addition, present study indicated that the levels of serum IL-2 in SLE patients were negatively correlated with SLEDAI (r = -0.5492, p < 0.0001) (**Figure 1C**). Next, the IL-2 mRNA levels in CD3^+^ T cells were detected and analyzed. However, there was no significant difference in IL-2 mRNA levels between SLE patients and healthy controls. (**Figure 1D**).

### Serum sIL-2Rα levels are elevated and correlated with IL-2 detection levels and SLEDAI in SLE patients

**Figure 2:**
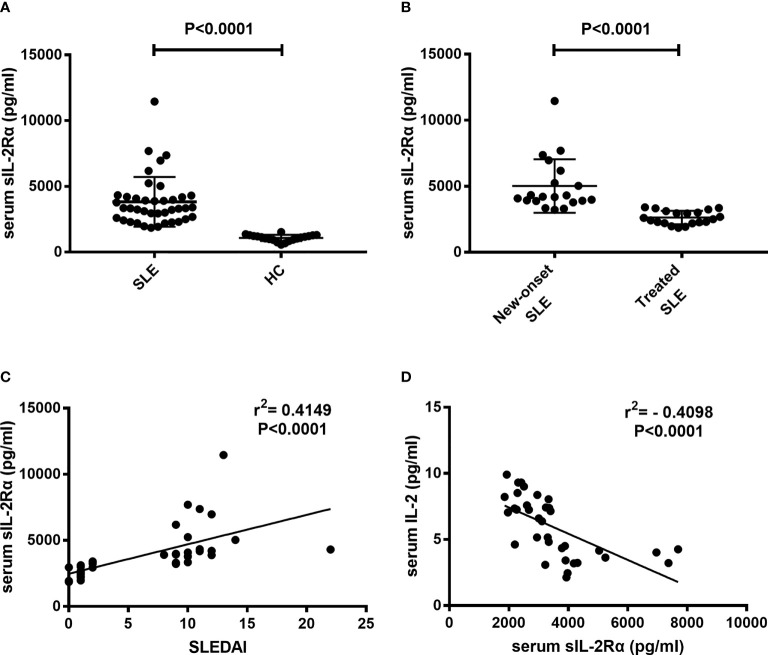
The levels of serum sIL-2Rα in SLE patients were elevated and correlated with the SLEDAI and serum IL-2 levels. **(A)** Serum sIL-2Rα levels in SLE patients were significantly higher than those in HCs. **(B)** Serum sIL-2Rα levels in new-onset SLE patients were significantly higher than those in treated SLE patients. **(C)** There was a positive correlation between serum sIL-2Rα and SLEDAI. **(D)** A negative correlation was observed between the levels of serum sIL-2Rα and serum IL-2 in SLE patients.

Sera were isolated from peripheral blood of SLE patients and healthy controls and detected for sIL-2Rα levels. Results showed that the levels of sIL-2Rα in SLE patients were significantly increased than those in HCs (3825 ± 299 pg/ml *vs*. 1082 ± 54 pg/ml, P<0.0001) (**Figure 2A**). The levels of sIL-2Rα in new diagnosed SLE patients were significantly increased than those in treated SLE patients (5020 ± 453 pg/ml *vs.* 2630 ± 114 pg/ml, P<0.0001) (**Figure 2B**). Furthermore, the correlations between the levels of sIL-2Rα, SLEDAI and the levels of IL-2 were analyzed. Results showed that the levels of sIL-2Rα in SLE patients were positively correlated with the SLEDAI (r = 0.4149, p < 0.0001) (**Figure 2C**), and negatively correlated with the levels of serum IL-2 (r = -0.4098, p < 0.0001) (**Figure 2D**).

### sIL-2Rα binds to IL-2 to form heterodimeric complex in peripheral blood of SLE patients

It has been reported that sIL-2Rα in human peripheral blood retains the ability to bind to IL-2, but there is no evidence to confirm the existence of sIL-2Rα/IL-2 complex in SLE patients. To verify the presence of sIL-2Rα/IL-2 complex in the peripheral blood of SLE patients, two special solid-phase sandwich ELISA were designed as described in method section. That is: Microplates pre-coated with anti-IL-2 antibody were matched with human IL-2R alpha biotinylated antibody, and the microplates pre-coated with anti-sIL-2Rα antibody were matched with human IL-2 biotinylated antibody, to detect sIL-2Rα/IL-2 complex in the serum of SLE patients and healthy controls (n=7). Some positive controls were made by mixing 12 pg/ml recombinant IL-2 with 5000 pg/ml sIL-2Rα at a volume ratio of 1:1, followed by incubating for 1 h to form heterodimeric complexes and detected by the mixed solid-phase sandwich ELISA system (n=7). In addition, exogenous IL-2 at the final concentration of 6 pg/ml and sIL-2Rα at the final concentration of 2500 pg/ml were added in serum from HC (n=7) and SLE patients (n=7), followed by incubating for 1 h and detected by the mixed solid-phase sandwich ELISA system (n=7). To confirm the feasibility of the detection systems, we verified the effectiveness and specificity of sIL-2Rα and IL-2 detection kits before the experiment.

**Figure 3:**
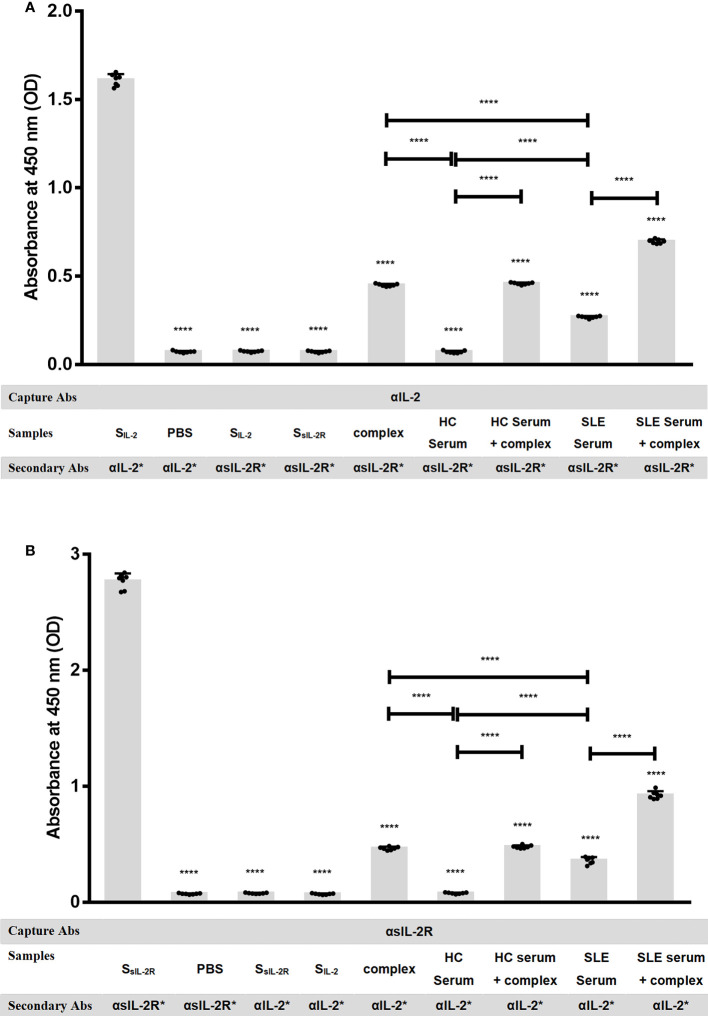
sIL-2Rα/IL-2 complex detection by mixed solid-phase sandwich ELISA. **(A)** Microplates pre-coated with anti-IL-2 antibody were used to detect heterodimeric sIL-2Rα/IL-2 complex in PBS, IL-2 standard, sIL-2Rα standard, serum of healthy controls, serum of SLE patients or heterodimeric complexes formed by pre-incubation of recombinant IL-2 (final concentration of 6 pg/ml) with sIL-2Rα (final concentration of 2500 pg/ml), in the condition of matching different labeled secondary antibodies (n=7). **(B)** Microplates pre-coated with anti-sIL-2Rα antibody were used to detect heterodimeric sIL-2Rα/IL-2 complex in PBS, sIL-2Rα standard, IL-2 standard, serum of healthy controls, serum of SLE patients or heterodimeric complexes formed by pre-incubation of recombinant IL-2 (final concentration of 6 pg/ml) with sIL-2Rα (final concentration of 2500 pg/ml), in the condition of matching different labeled secondary antibodies (n=7). Data were expressed as mean ± standard deviation (SD) of at least three independent experiments. Statistical significance was determined by one-way analysis of variance (ANOVA) followed by *post hoc* test. SLE, systemic lupus erythematosus; HCs, healthy controls; αIL-2: anti-IL-2 antibody; αsIL-2Rα: anti-sIL-2Rα antibody; S_IL-2_, standard of IL-2; S_sIL-2Rα_, standard of sIL-2Rα; complex, heterodimeric complexes formed by pre-incubation of recombinant IL-2 with sIL-2Rα. *: labeled. “NS” means no significance; *p < 0.05; ****p < 0.0001.

The results demonstrated that when microplates pre-coated with anti-sIL-2Rα or anti-IL-2 antibody were matched with corresponding labeled secondary antibodies both sIL-2Rα and IL-2 ELISA systems can effectively detect standard of corresponding target protein, indicating that these two detection systems are effective. However, when the microplates pre-coated with anti-sIL-2Rα antibody were matched with labeled anti-IL-2 antibody, both sIL-2Rα standard and IL-2 standard could not be detected. It demonstrated that the microplates pre-coated with anti-sIL-2Rα antibody cannot capture IL-2 in the sample, and the labeled anti-IL-2 antibody will not cross react with sIL-2Rα. Similarly, when the microplates pre-coated with anti-IL-2 antibody were matched with labeled anti-sIL-2Rα antibody, both IL-2 standard and sIL-2Rα standard could not be detected. It demonstrated that the microplates pre-coated with anti-IL-2 antibody cannot capture sIL-2Rα in the sample, and the labeled anti-sIL-2Rα antibody will not cross react with IL-2 (**Figure 3**). However, the mixed solid-phase sandwich ELISA system, both solid-phase anti-sIL-2Rα antibody matched with labeled anti-IL-2 antibody and solid-phase anti-IL-2 antibody matched with labeled anti-sIL-2Rα antibody, can detection signal of heterodimeric sIL-2Rα/IL-2 complex in the serum of SLE patients, but not in the serum from healthy volunteers. And, when the concentration of sIL-2Rα was greater than or equal to 1250pg/ml in the positive controls, the hybrid solid-phase sandwich ELISA system could detect the signal of heterodimeric sIL-2Rα/IL-2 complex. Furthermore, simultaneous addition of exogenous IL-2 and sIL-2Rα in serum can significantly improve the detection signal of heterodimeric sIL-2Rα/IL-2 complex (**Figure 3**).

### sIL-2Rα leads to a false reduction in the detection level of IL-2

**Figure 4:**
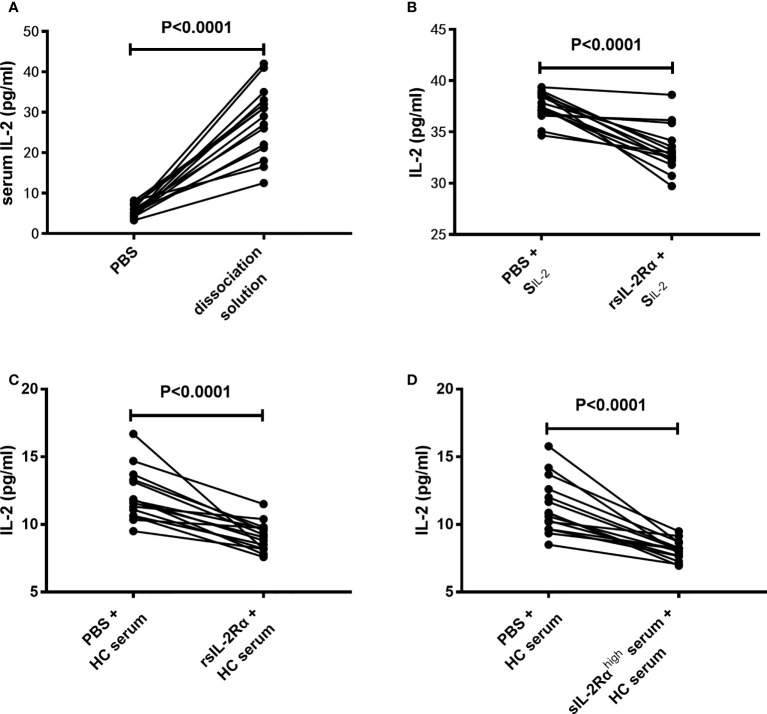
Effects of sIL-2Rα on the detection level of IL-2. **(A)** The serums of SLE patients were pretreated with immune complex dissociation solution or equal volume of PBS at 37°C for 30 min and detected for IL-2 levels by ELISA. **(B)** IL-2 standards were co-incubated with recombinant sIL-2Rα or equal volume of PBS for 1 h and detected for IL-2 levels by ELISA. **(C)** Serums of healthy controls were co-incubated with recombinant sIL-2Rα or equal volume of PBS for 1 h and detected for IL-2 levels by ELISA. **(D)** Serums of healthy controls was mixed with serums from new onset SLE patients with high levels of sIL-2Rα or equal volume of PBS for 1 h and detected for IL-2 levels by ELISA. S_IL-2_ standard of IL-2; rsIL-2Rα recombinant sIL-2Rα.

Considering the fact that the IL-2 mRNA levels in CD3^+^ T cells have no significant difference between SLE patients and HCs, and sIL-2Rα in peripheral blood of SLE patients is produced in large quantities and can combine with IL-2 to form immune complex, we speculate that whether the decrease of serum IL-2 level in SLE patients is due to the false decrease of IL-2 detection caused by the formation of sIL-2Rα/IL-2 complex. To test this hypothesis, the serum from SLE patients was pretreated with immune complex dissociation solution or equal volume of PBS, followed by detection for IL-2 levels by ELISA. The results showed that pretreatment with immune complex dissociation solution significantly elevated the detection levels of IL-2 than pretreatment with PBS (**Figure 4A**).

To further confirm the influence of sIL-2Rα on IL-2 detection, recombinant human sIL-2Rα and serum samples with high concentration of sIL-2Rα were used to co-incubate with IL-2 standard or serum from HC, followed by detection for IL-2 levels in the mixed solution or serum by ELISA. Results demonstrated that the detection levels of IL-2 in IL-2 standard were significantly decreased when exogenous sIL-2Rα were added (**Figure 4B**). In addition, the detection levels of IL-2 in HC serum were significantly decreased when exogenous sIL-2Rα or sera with high concentration of sIL-2Rα were added (**Figures 4C, D**).

### sIL-2Rα leads to a significant decrease of IL-2 bioactivity

**Figure 5:**
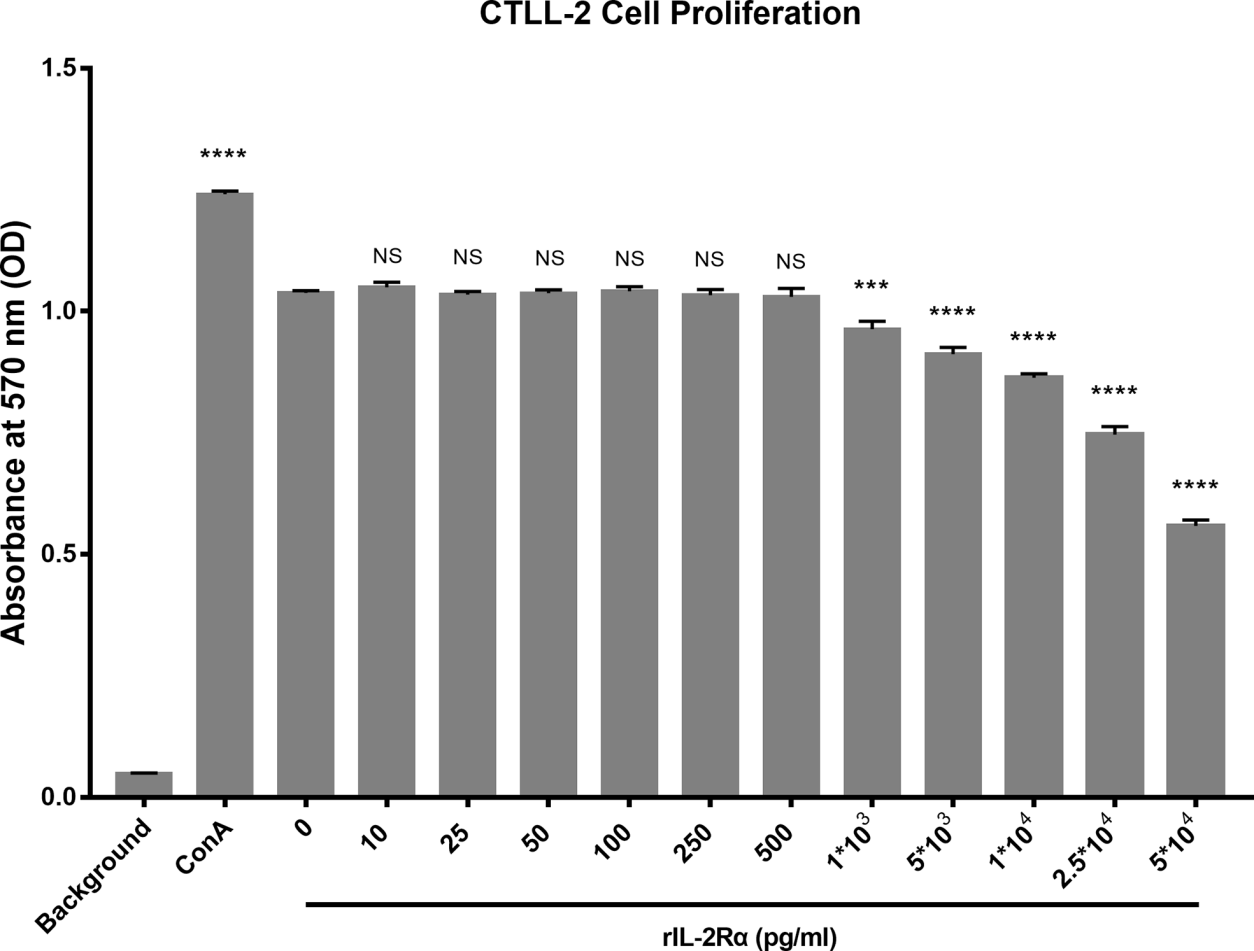
sIL-2Rα suppressed the proliferation of CTLL-2 cells *in vitro*. CTLL-2 cells were cultured in medium containing IL-2 at the concentration of 6 pg/ml, with the addition of sIL-2Rα at different concentration or Con A (10 μg/ml) for 18 h. Control wells containing no cells or CTLL-2 cells without IL-2 were designed as background and negative control. The cell proliferation was measured by MTT assay. Data were expressed as mean ± standard deviation (SD) of at least three independent experiments. Statistical significance was determined by one-way analysis of variance (ANOVA) followed by *post hoc* test. P value versus the negative control. “NS” means no significance; *p < 0.05; ***p < 0.001; ****p < 0.0001.

Aforementioned research demonstrated that increased sIL-2Rα in SLE patients could bind to IL-2 and leads to a false reduction in the detection level of IL-2. In order to investigate whether sIL-2Rα can also inhibit the biological activity of IL-2, the proliferation of CTLL-2 cells was detected in the presence of IL-2 (6 pg/ml), with or without the addition of sIL-2Rα. Results showed that sIL-2Rα suppressed the proliferation of CTLL-2 cells in a dose dependent manner (**Figure 5**). When the concentration of sIL-2Rα was 1×10^3^, 5×10^3^, 1×10^4^, 2.5×10^4^, and 5×10^4^ pg/ml, the cell inhibition rate was 7.49%, 12.75%, 17.71%, 29.55%, and 48.48%, respectively. Therefore, the concentration of sIL-2Rα was 5×10^4^ pg/ml when the inhibition rate reached about 50%.

### sIL-2Rα leads to the obstruction of Treg differentiation

**Figure 6:**
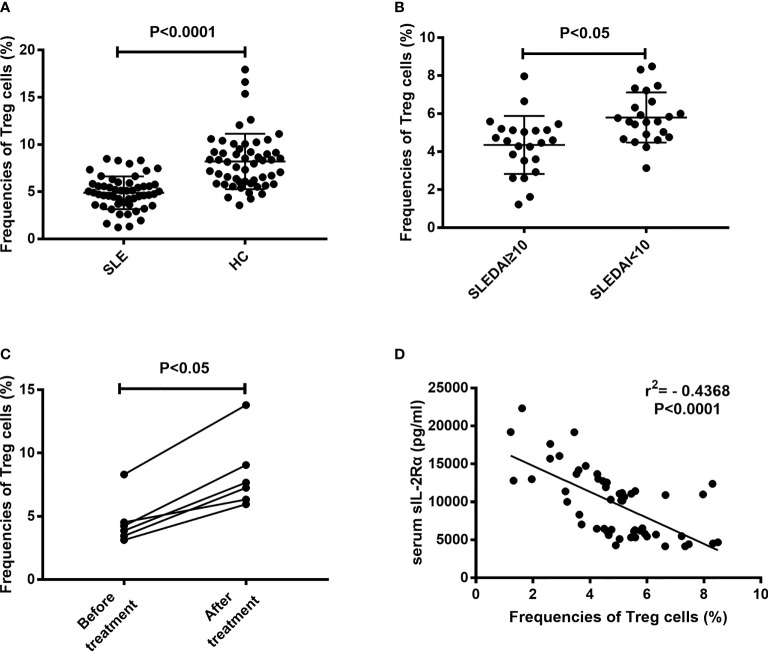
Correlation between the frequencies of Treg cells and serum sIL-2Rα levels. **(A)** The frequencies of peripheral CD4^+^CD25^+^CD127^low/-^ Treg cells were significantly lower in SLE patients than those in HCs. **(B)** The frequencies of peripheral CD4^+^CD25^+^CD127^low/-^ Treg cells were significantly lower in active SLE patients than those in patients with inactive SLE. **(C)** The frequencies of peripheral CD4^+^CD25^+^CD127^low/-^ Treg cells were increased after effective therapy in SLE patients. **(D)** An inverse correlation between sIL-2Rα levels and the frequencies of peripheral CD4^+^CD25^+^CD127^low/-^ Treg cells was shown.

An important feature of immune cell differentiation in SLE patients is that the differentiation of regulatory T (Treg) cells is obstructed. The mechanism for this phenomenon is considered to be related to the decrease of IL-2 level in SLE patients. The above results show that sIL-2Rα can suppress the bioactivity of IL-2, so we speculate whether sIL-2Rα is involved in the differentiation regulation of Treg cells. Our results confirmed that the frequencies of peripheral CD4^+^CD25^+^CD127^low/-^ Treg cells were significantly lower in SLE patients than those in healthy controls (**Figure 6A**). The frequencies of Treg cells were significantly lower in active SLE patients (SLEDAI≥10) than those in SLE patients with stable disease (SLEDAI<10) (**Figure 6B**). Furthermore, we found that the frequencies of circulating Treg cells in new onset SLE patients were significantly increased after effective therapy (**Figure 6C**). In addition, our results showed that the frequencies of circulating Treg cells were inversely correlated with the levels of serum sIL-2Rα in SLE patient (**Figure 6D**).

**Figure 7:**
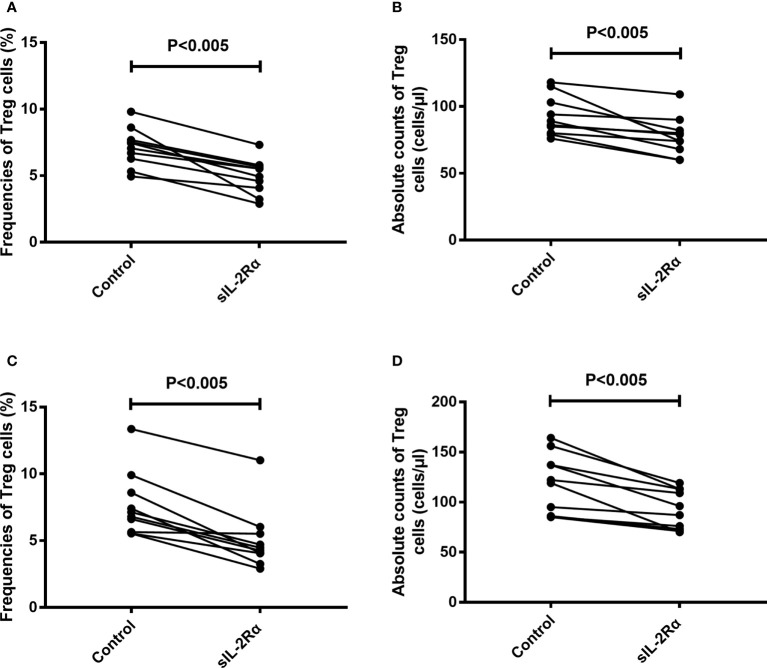
sIL-2Rα suppresses the differentiation of Treg cells. **(A, B)** Heparin anticoagulant whole blood was collected from healthy volunteers and cultured in 37°C and 5% CO_2_ for 96 h, with or without the addition of recombinant sIL-2Rα at the final concentration of 1000 pg/ml. **(C, D)** Heparin anticoagulant whole blood was collected from healthy volunteers, stimulated with PHA (10 μg/ml) and cultured in 37°C and 5% CO_2_ for 96 h, with or without the addition of recombinant sIL-2Rα at the final concentration of 1000 pg/ml. The frequencies and absolute counts of peripheral CD4^+^CD25^+^CD127^low/-^ Treg cells in CD4^+^ cells were detected by FCM.

To further confirm the relation between sIL-2Rα and the differentiation of Treg cells, freshly isolated peripheral blood from healthy volunteer were cultured for 96 h with or without the addition of exogenous sIL-2Rα, followed by detection for the frequencies and absolute counts of Treg cells. Results showed that exogenous addition of sIL-2Rα significantly decrease the frequencies and absolute counts of Treg cells, whether or not PHA was used to activate the T cells (**Figure 7**).

## Discussion

SLE is a multi-organ autoimmune disease characterized by chronic and repeated activation of the immune system, severe inflammation, and organ damage (22). One of the main characteristics of SLE patients is the imbalance of immune regulation, mainly manifested in the decrease of the frequencies of Treg cells and the increase of the frequencies of effector T cells (11). Among them, the decrease of Treg cell frequency and function is one of the important mechanisms leading to the compromise of peripheral immune tolerance and the activation of immune response against autoantigens in SLE patients (5, 6). Recent studies have found that the decline of the frequency of Treg cells in SLE patients is mainly due to the decrease of IL-2 level and function (23).

IL-2 is mostly produced by CD4^+^ T helper cells (TH) in secondary lymphoid organs, and is also produced by CD8^+^ T cells, natural killer cells (NK) and natural killer T cells (NKT) at a lower level (7, 24). Previous studies have reported that the amount of IL-2 produced by T cells from patients and mice with SLE is reduced, which is thought to contribute to increased susceptibility to infection, reduced activation-induced cell death, and subsequent prolonged survival of autoreactive lymphocytes (3).

IL-2 is a multifunctional cytokine produced mainly by activated T cells, which has essential roles in regulating the activation and differentiation of T cells (25). This cytokine was first considered to be a T cell growth factor because of its important roles in the generation and maintenance of an effective T cell response (7). However, recently another crucial role of IL-2 in the proliferation, function and homeostasis of Treg cells is considered to be its main nonredundant function (9). Therefore, the decline of IL-2 level, or suppression of IL-2 activity will lead to the compromise of peripheral tolerance and promotion of immune response.

It has long been found that the level of serum IL-2, mainly detected by ELISA, in SLE patients is significantly lower than that of healthy people (26). Moreover, recent studies have confirmed that the decline of IL-2 level is one of main mechanisms for the imbalance of immune regulation in SLE patients, which is thought to contribute to impairment of peripheral tolerance, reduced activation-induced cell death, and subsequent prolonged survival of autoreactive lymphocytes (27). Further researches based on this finding showed that injection of recombinant IL-2 can significantly alleviate disease symptoms in both SLE patients and lupus mice (28). These studies demonstrated that the injection of recombinant IL-2 can significantly expand the number of Treg cells and suppress the differentiation and function of self reactive effector T cells (12). It is suggested that exogenous supplement of IL-2 can correct the immune imbalance caused by the decline of IL-2 and achieve the purpose of treating SLE. In recent years, the preliminary results of some clinical trials have confirmed the effect of IL-2 supplementation scheme in the treatment of SLE (28, 29). However, as a biological agent, the direct use of IL-2 as a drug to therapy SLE has been problematic in some extent (30). First, its short half-life (15-30 min) makes patients need to receive injection for a long time and frequently. Secondly, as a multifunctional cytokine, exogenous IL-2 has many toxic and side effects when applied to human body, such as liver and kidney injury. Although the toxicity of IL-2 can be reduced by reducing the dosage, but it is hard to avoid. The possibility of production of Anti-IL-2 caused by multiple injections is also an issue to be considered for long-term application. Therefore, preventing or reversing the decline of IL-2 in SLE patients may be a safer and more effective strategy for SLE treatment.

In this study, our results showed that the serum IL-2 level of SLE patients detected by ELISA is significantly lower than that of healthy people, which is consistent with the results in the literatures (26). However, our study found that the level of IL-2 mRNA in CD3^+^ T cells of SLE patients was not significantly down regulated compared with healthy people. Previously, a study demonstrated that except the naïve CD4+ T cell population, the frequencies of IL-2 producing CD4+ T cells had no statistical difference between SLE patients and healthy subjects (31). This study did not establish any correlation between the frequency of IL-2 producing cells and SLEDAI. Both this study and our results suggest that the ability of PBMCs to express IL-2 in SLE patients is not significantly down regulated, there may be some other mechanisms that affect the detection level and function of IL-2 in SLE patients.

As a cytokine, IL-2 needs to bind to its specific receptor, IL-2R, before it can transmit its signal to the target cells, and then exert its biological effect (32). IL-2R is composed of three distinct glycopeptide subunits called α (IL-2Rα, CD25), β (IL-2Rβ, CD122) and γ (IL-2Rγ, CD132) chain (7). Although each subunit of membrane-bound IL-2R is able to bind IL-2 independently, they have only low or intermediate affinity. It has been demonstrated that the high-affinity IL-2R consists of all three subunits. Generally, Treg cells constitutively express high affinity IL-2 receptors (IL-2RαβƳ), which is 100 folds higher affinity than low or intermediate affinity receptor (33). Therefore, Treg cell is very sensitive to the signal of IL-2. This may be the main reason for the down-regulation of Treg cell frequency in SLE patients. Except for Treg cells, most non-stimulated peripheral blood lymphocytes do not express α chain, thus express only low or intermediate affinity IL-2R (34). However, IL-2Rα is rapidly induced and expressed in large quantities on T cells after the activation of mononuclear cells. It was also found that activated T cells produced soluble forms of IL-2Rα, namely sIL-2Rα or sCD25. Studies demonstrated that sIL-2Rα generated as a result of proteolytic cleavage of membrane sIL-2Rα on activated T cells (35, 36).

Studies have shown that sIL-2Rα retains the ability to bind IL-2, so it may inhibit the function of membrane sIL-2Rα through competition (37). However, some studies suggest that serum sIL-2Rα may also stabilize IL-2 and prolong the half-life of IL-2 by forming a complex with IL-2 (15). However, its role in SLE patients has not been reported. Although it has been found for a long time that the level of serum sIL-2Rα in SLE patients is increased, there is no direct evidence to confirm the existence of sIL-2Rα/IL-2 complex in peripheral blood of SLE patients. In this study, by using two mixed solid-phase sandwich ELISA, we confirmed the existence of sIL-2Rα/IL-2 complex in the peripheral blood of SLE patients for the first time. At the same time, this study also found that the levels of sIL-2Rα were inversely proportional to the levels of serum IL-2 in patients with SLE. Further, by mixing sIL-2Rα with IL-2 standard or human serum, we found for the first time that sIL-2Rα would significantly reduce the detection level of IL-2. Moreover, pretreatment with immune complex dissociation solution containing surfactant can significantly up-regulate the detection level of serum IL-2 in SLE patients, which also confirms the existence of sIL-2Rα/IL-2 complex from another side. These results suggested that sIL-2Rα can obstruct the detection of IL-2 by combining with IL-2, resulting in a false decrease in the detection level of IL-2. Although further research is needed, we speculate that this effect may be related to the occlusion of IL-2 epitope after the formation of sIL-2Rα/IL-2 complex.

After clarifying the effect of sIL-2Rα on the detection level of IL-2, we further investigated the effect of sIL-2Rα on the biological function of IL-2. Our results show that sIL-2Rα can significantly inhibit the proliferation of CTLL-2 cells induced by IL-2, indicating that sIL-2Rα can not only interfere with the detection of IL-2, but also inhibit the biological function of IL-2. IL-2 is the main cytokine supporting Treg survival and differentiation. Next, we investigated the relationship between the levels of serum sIL-2Rα and the frequencies of Treg cells in SLE patients. The results showed that they had a significant negative correlation. Further investigation found that exogenous addition of sIL-2Rα to human peripheral blood can significantly suppress the differentiation of Treg cells, leads to the decline of Treg cell frequency and absolute count. This suppression was more significant when PHA was used to activate T cells. These results thus proved that sIL-2Rα can significantly suppress the differentiation of Treg cells in SLE patients.

In summary, this study confirmed that the levels of serum sIL-2Rα in SLE patients were increased significantly and closely related to the disease activity of SLE patients. Moreover, our study demonstrated for the first time that sIL-2Rα in peripheral blood of SLE patients can combine with IL-2 to form a complex, decrease the detection level and biological activity of IL-2, lead to the suppression of the differentiation of Treg cells and compromise of peripheral tolerance. Our study therefore suggests that the elevated sIL-2Rα in peripheral blood of SLE patients may be related to the imbalance of immune regulation in SLE patients and may be involved in the occurrence and development of SLE. Therefore, sIL-2Rα may be a promising target for the treatment of SLE in the further.

## Data availability statement

The raw data supporting the conclusions of this article will be made available by the authors, without undue reservation.

## Ethics statement

The studies involving human participants were reviewed and approved by the Ethics Committee of The First Affiliated Hospital of Nanchang University [approval no. (2022)CDYFYYLK(06-005)]. The patients/participants provided their written informed consent to participate in this study.

## Author contributions

JL and QL designed the experiments. SY, LZ, and YR collected clinical data. Experimental works were performed by DL and ZH, and data were analyzed by SY, YG, SX, DL, and ZH. DL prepared the manuscript. All authors contributed to the article and approved the submitted version.

## Funding

This work was supported by the grants from the National Natural Science Foundation of China (Grant No. 82160308), the project for high end talent of Science and Technology Innovation in Jiangxi “double thousand plan” (Grant No. jxsq2019201024), the Project for Academic and Technical Leaders of Major Disciplines of Jiangxi Province (Grant No. 20172BCB22026), the Natural Science Foundation of Jiangxi Province of China (Grant No. 20212BAB216028).

## Acknowledgments

We thank the patients and healthy volunteers who participated in this study.

## Conflict of interest

The authors declare that the research was conducted in the absence of any commercial or financial relationships that could be construed as a potential conflict of interest.

## Publisher’s note

All claims expressed in this article are solely those of the authors and do not necessarily represent those of their affiliated organizations, or those of the publisher, the editors and the reviewers. Any product that may be evaluated in this article, or claim that may be made by its manufacturer, is not guaranteed or endorsed by the publisher.
